# A *Mec17-Myosin II* Effector Axis Coordinates Microtubule Acetylation and Actin Dynamics to Control Primary Cilium Biogenesis

**DOI:** 10.1371/journal.pone.0114087

**Published:** 2014-12-10

**Authors:** Yanhua Rao, Rui Hao, Bin Wang, Tso-Pang Yao

**Affiliations:** Department of Pharmacology and Cancer Biology, Duke University, Durham, North Carolina, United States of America; Zhejiang University, China

## Abstract

Primary cilia are specialized, acetylated microtubule-based signaling processes. Cilium assembly is activated by cellular quiescence and requires reconfiguration of microtubules, the actin cytoskeleton, and vesicular trafficking machinery. How these components are coordinated to activate ciliogenesis remains unknown. Here we identify the microtubule acetyltransferase *Mec-17* and myosin II motors as the key effectors in primary cilium biogenesis. We found that myosin IIB (*Myh10*) is required for cilium formation; however, myosin IIA (*Myh9*) suppresses it. *Myh10* binds and antagonizes Myh9 to increase actin dynamics, which facilitates the assembly of the pericentrosomal preciliary complex (PPC) that supplies materials for cilium growth. Importantly, *Myh10* expression is upregulated by serum-starvation and this induction requires *Mec-17*, which is itself accumulated upon cellular quiescence. Pharmacological stimulation of microtubule acetylation also induces *Myh10* expression and cilium formation. Thus cellular quiescence induces *Mec17* to couple the production of acetylated microtubules and *Myh10*, whose accumulation overcomes the inhibitory role of *Myh9* and initiates ciliogenesis.

## Introduction

Primary cilia are evolutionarily conserved, microtubule-based organelles critical for detecting and transmitting mechanical and chemical cues. The biological functions of primary cilia have long been overlooked until the discovery of a cohort of cilia-related human developmental disorders, including Bardet–Biedl syndrome (BBS) [1], Joubert syndrome [2] and Merkel-Gruber syndrome [3]. Human genetic studies in combination with biochemical and cell biological approaches have identified the basic components and mechanisms underlying primary cilium formation and function [4], [5]. When ciliogenesis is initiated upon cellular quiescence, the mother centriole translocates to the cortical plasma membrane and forms the basal body, from where the ciliary microtubules are polymerized and form the axoneme. In coordination with axoneme growth, specialized vesicles become concentrated around the basal body and provide new membranes and proteins to support cilium growth [6]. Disruption of this pericentrosomal preciliary compartment (PPC), which is enriched for *Rab11* positive recycling endosomes and proteins important for membrane fusion and transport including *Rab8*, *PCM-1*, and *Cep290*, leads to defects in cilia formation [6]–[8]. The coordination of PPC assembly with microtubule-axoneme growth is thus critical for ciliogenesis but its molecular basis remains poorly understood.

Primary cilium formation requires the reorganization of cellular cytoskeleton, particularly microtubules which provide both structural components and intraflagellar transport [9]. One salient feature of ciliary microtubules is the prevalent acetylation on lysine (K)-40 of α-tubulin [10]. The presence of acetylated microtubules, in fact, is the most commonly used marker for primary cilia although its exact function in the cilium remains uncertain. α-tubulin acetylation is primarily controlled by the acetyltransferase *Mec-17* (also termed *alphaTAT1*) [11], [12] and the deacetylase *HDAC6* [13]. *Mec-17* knockdown does not eliminate primary cilium formation [14], [15]; however it disrupts the normal kinetics of cilium biogenesis [12]. On the other hand, *HDAC6* has been proposed to facilitate primary cilium resorption [16]. While these findings suggest a regulatory role of microtubule acetylation in primary cilium formation, how the production of acetylated microtubules is coupled to ciliogenesis is not known.

In addition to microtubules, several components of the actin cytoskeleton were recently identified as cilium regulators [7]. The analyses of these factors have revealed a general inhibitory role of the actin cytoskeleton in ciliogenesis where stable actin cytoskeleton prevents the formation of PPC enriched for Rab11-positive recycling endosomes [7]. Thus, reorganization of the actin network is required for efficient delivery of membranes and materials for cilium growth. Supporting this view, microRNA-129-3p, a positive regulator for cilium formation, targets genes involved in the formation of a branched actin network [17]. The mechanism by which quiescent cells release the inhibitory brake enforced by the actin network to activate ciliogenesis remains to be characterized.

In this report, we provide evidence that non-muscle myosin IIA and IIB and the tubulin acetyltransferase *Mec-17* form the central molecular circuit that controls cilium formation. We show that myosin IIB (*Myh10*) promotes, whereas IIA (*Myh9*) inhibits, ciliogenesis. The opposing activity of Myh10 and Myh9 is mediated through the actin dynamics, which in turn controls PPC assembly. We found that Myh10 expression is positively regulated by the tubulin acetyltransferase *Mec-17*. Importantly, both *Mec-17* and *Myh10* gene expression are induced by serum starvation conditions that activate cilium formation. In *Mec-17*-deficient cells subject to serum starvation, *Myh10* is not induced and ciliogenesis is deregulated. Conversely, pharmacological inhibition of HDAC6 increases microtubule acetylation, *Myh10* expression and cilium formation. Our results suggest that *Mec-17*-dependent microtubule acetylation is coupled to the induction of Myh10, whose accumulation overcomes Myh9-dependent actin cytoskeleton stabilization and promote primary cilium assembly.

## Results

### *Myh10* Is Required for Cilium Formation

**Figure 1:**
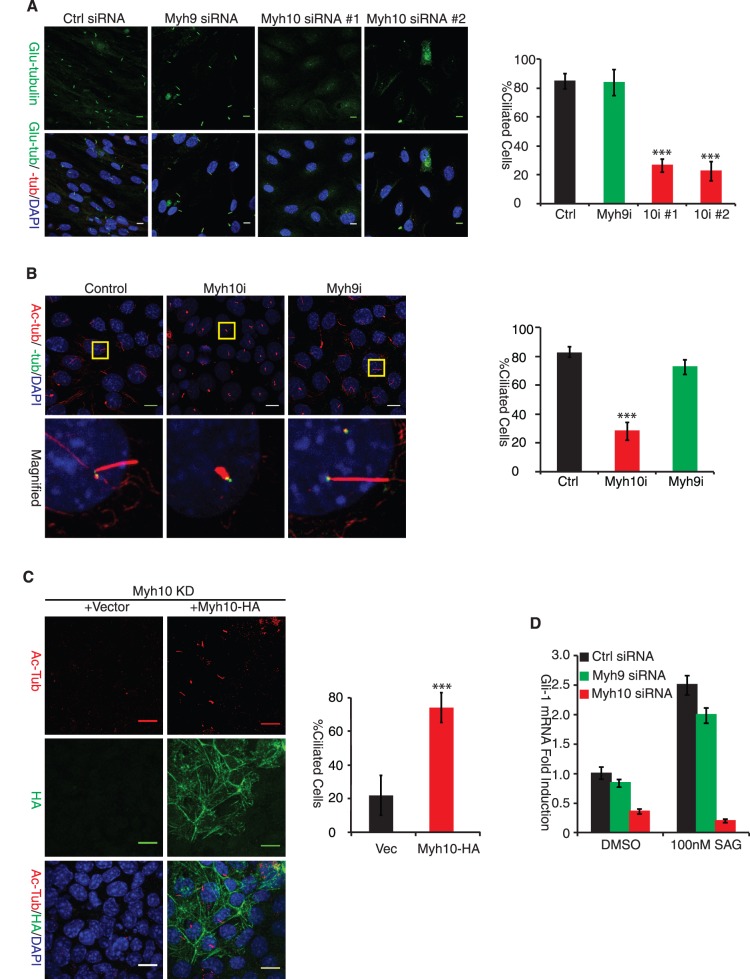
Myh10 Is Required for Ciliogenesis. (A) Myh10 knockdown inhibits cilium formation in RPE-Mchr1^GFP^ cells. RPE-Mchr1^GFP^ cells were transfected with control siRNA (left panel), Myh10 siRNA #1 and #2 (middle panels) and Myh9 siRNA duplex (right panel) and then serum starved for 36 hours. Cells were fixed by methanol for indirect fluorescence staining using rabbit anti-glu-tubulin (pseudo colored with green) and mouse anti-γ-tubulin (red). Nuclei were counter stained with DAPI. Scale bar: 10 µm. Percentage of ciliated cells (acetylated tubulin staining longer than 1 µm) were quantified under confocal microscopy (right panel). Error bars represent standard deviations (s.d.) from triple replicates. ***, t-test p<0.001. (B) Myh10 is required for ciliogenesis in IMCD3 cells. IMCD3 cells were transfected with siRNA duplexes targeting Myh9 or Myh10 (left panel) and were allow to grow confluent for 72 hours after transfection. Cells were methanol fixed and immunostained with mouse anti-acetylated tubulin (red) or rabbit anti-γ-tubulin (green) to label cilia and centrosomes respectively. Yellow insets were magnified to allow better visualization of representative primary cilium images in each knockdown group (left panel, bottom). Percentage of ciliated IMCD3 cells from each knockdown group was quantified under the confocal microscopy (right panel). Error bars represent standard deviations (s.d.) from triple replicates. ***, t-test p<0.001. (C) Human Myh10 overexpression restored ciliogenesis in Myh10-knockdown IMCD3 cells. IMCD3 cells stably expressing MSCV-Puro vector (left panel, left) or MSCV-Myh10-HA (left panel, right) were transfected with Myh10 siRNA duplex, methanol fixed and stained for acetylated tubulin (red) and HA-tag (green) 72 hours after transfection. Percent of ciliated cells was quantified and shown in right panel. Error bar represents standard deviation (s.d.) from triple replicates. ***, t-test p<0.001. Scale bar: 10 µm. (D) Myh10 knockdown abrogates cilia-mediated sonic hedgehog signaling. RPE-Mchr1^GFP^ cells were transfected with control (black), Myh9 (green) and Myh10 (red) siRNA and serum starved (DMEM with 0.2% FBS) for 48 hours to induce ciliogenesis. Cells were then stimulated with 100 nM SAG for 24 hours. Total mRNA from each group was collected for real-time PCR analysis of Gli1 mRNA expression. Error bar represents standard deviation (s.d.) of triple biological replicates.

To investigate the role of the actin network in ciliogenesis, we focused on non-muscle myosin II motors, which are known to regulate actin network dynamics and actinomyosin-microtubule crosstalk [18], [19]. We adopted the serum starvation protocol to induce cilium formation in RPE-Mchr1^GFP^ cells, a retina pigment epithelial cell line expressing GFP-fused melanin-concentrating hormone receptor 1 (*Mchr1*) protein [20]. To study the role of myosin IIA (*Myh9*) and IIB (*Myh10*) in cilium formation, we first designed siRNA duplexes specifically targeting *Myh10* or *Myh9* mRNA. Western blot analysis and Q-PCR confirmed the effective knockdown of *Myh10* and *Myh9* expression by siRNAs (S1A Figure). When RPE-Mchr1^GFP^ cells were subjected to serum deprivation for 24 hours to induce ciliogenesis, *Myh9* siRNA-treated cells were able to form cilia as efficiently as control siRNA group (Fig. 1A). In stark contrast, two different *Myh10* siRNA duplexes both dramatically inhibited cilium formation (Fig. 1A). Knocking down *Myh10*, but not *Myh9*, in mouse IMCD3 cells also potently inhibited cilium formation in IMCD3 cells (Fig. 1B and Fig. S1B). Moreover, re-introducing a wild type human *Myh10* restored cilium formation in *Myh10*-knockdown (KD)-IMCD3 cells (Fig. 1C). These findings show that *Myh10* is required for efficient ciliogenesis. It is also worthwhile to point out that *Myh10* knockdown does not stop cells from proliferating before reaching confluency in our experiments, suggesting the effect of *Myh10* on ciliogenesis is not an indirect consequence of altered cell cycle.

Cilia are important for sonic hedgehog signaling [21]. To confirm that *Myh10* is required for cilia-mediated signal transduction, we stimulated cells with SAG [21], a small molecule agonist of Smoothened, to induce sonic hedgehog signaling in serum-starved cells. We measured *Gli-1* mRNA induction, a downstream target of hedgehog signaling, by RT-PCR. As shown in Fig. 1D, *Myh10* (red bar) but not *Myh9* (green bar) knockdown strongly inhibited *Gli-1* induction upon SAG stimulation, indicating that *Myh10* is required for cilium-mediated sonic hedgehog signal transduction. These experiments collectively demonstrated that *Myh10* is required for proper ciliogenesis.

### *Myh10* Antagonizes *Myh9* Activity in Cilium Formation

**Figure 2:**
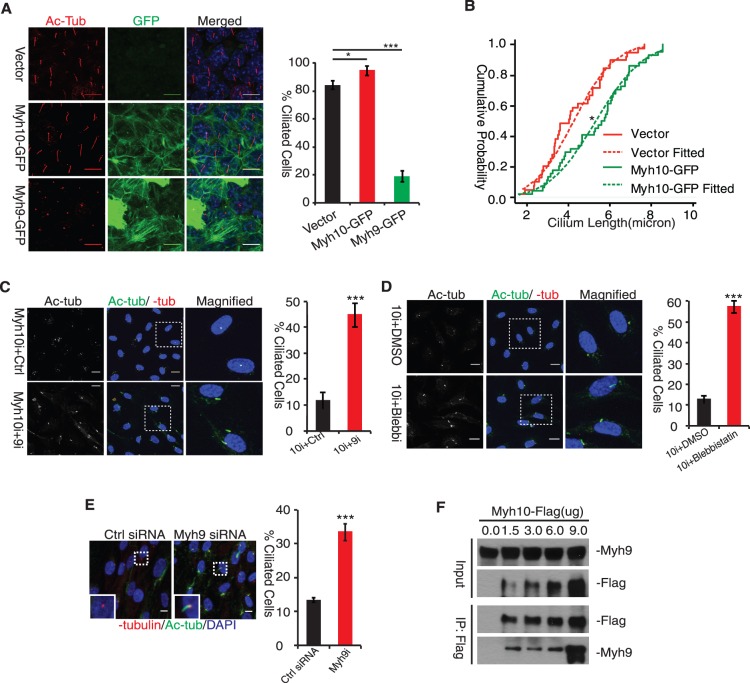
Myh10 Counteracts Myh9 Activity to Promote Ciliogenesis. (A) Myh10 and Myh9 displayed opposing activity on ciliogenesis. IMCD3 cells were stably transfected with pCR3.1 vector (left panel, upper row), CMV-Myh9-GFP (left panel, bottom row) and CMV-Myh10-GFP (left panel, middle row) and grew to confluence to induce ciliogenesis. Cells were fixed and stained with acetylated tubulin antibody to label cilia. Percent of cells with cilia longer than 1 µm were counted and plotted (upper right panel). Scale bars: 10 µm. Error bars represents standard deviations (s.d.) from triple replicates. ***, t-test, p<0.001. (B) Myh10 overexpression promotes cilia elongation. Cilia length in vector and Myh10-GFP cells was plotted (lower right panel) as cumulative probability distribution curve (solid line) and fitted with normal distribution function curve (dash line). *, t-test p<0.01. (C) Myh9 knockdown partially rescued cilia defects in Myh10 knockdown cells. Myh10 siRNA duplex transfected RPE-Mchr1^GFP^ cells were transfected with either control (left panel, upper row) or Myh9 (left panel, bottom row) siRNA then serum starved to induce ciliogenesis. Percent of ciliated cells was quantified and shown in right panel. White insets were magnified to allow better visualization of representative primary cilia (left panel, right column). Scale bars: 10 µm. Error bars represents standard deviations (s.d.) from triple replicates. ***, t-test p<0.001. (D) Blebbistatin treatment partially restored cilia defects in Myh10 knockdown cells. Myh10-knockdown RPE-Mchr1^GFP^ cells were treated with either DMSO or 25 µM blebbistatin when inducing ciliogenesis. Percent of ciliated cells was quantified and shown in right panel. White insets were magnified to allow better visualization of representative primary cilia (left panel, right column). Scale bars: 10 µm. Error bars represents standard deviations (s.d.) from triple replicates. ***, t-test p<0.001. (E) Myh9 knockdown promoted spontaneous ciliogenesis. Control and Myh9-knockdown RPE-Mchr1^GFP^ cells were switched to DMEM with 5% FBS for 24 hours then fixed and labeled with acetylated tubulin (green) and γ-tubulin (red) antibody to visualize cilia and basal body. Percent of ciliated cells was quantified (right panel). Scale bars: 10 µm. Error bars represents standard deviations (s.d.) from triple replicates. ***, t-test, p<0.001. (F) Myh9 physically interacts with Myh10. HEK293-T cells were transfected with different amount of MSCV-Flag-HA-Myh10 plasmids (0, 1.5, 3.0, 6.0, 9.0 ug) and collected for immunoprecipitation using M2-Flag affinity gel. Precipitates were analyzed for Myh10-Flag and Myh9.

To further ascertain the role of *Myh10* in cilium formation, we determined whether an increase of *Myh10* expression is sufficient to promote the growth of cilia. To this end, we stably expressed *Myh10*-*GFP* in IMCD3 cells. *Myh9-GFP* [22] was included as a control. We then labeled acetylated tubulin to examine cilium formation in these two stable cell lines. *Myh10-GFP* IMCD3 cells form cilia at a modestly higher percentage than control cells (Fig. 2A, left panel, middle row, and right panel, red bar); however, cilium length in *Myh10-GFP* expressing cells is significantly longer (Fig. 2B). This data indicate that increasing *Myh10* expression stimulates cilia elongation. Unexpectedly, cilium formation was dramatically inhibited in *Myh9-GFP* expressing IMCD3 cells (Fig. 2A, left panel, bottom row, and right panel, green bar). Thus *Myh9*, in contrast to *Myh10*, appears to be a negative regulator of cilium formation.

As overexpression of Myh9 and Myh10 caused opposite phenotypes on cilia morphology, we tested if Myh10 promotes cilium formation by antagonizing Myh9 activity, as these two myosin motors can form hetero-oligomers (Fig. 2F). To test this possibility, we knocked down both *Myh9* and *Myh10* expression by siRNA in RPE-Mchr1^GFP^ cells and assessed cilium formation. In contrast to *Myh10* single knockdown, where few cilia were observed, concurrent knockdown of *Myh9* restored cilium formation in more than 40% of the cells (Fig. 2C). Further supporting the conclusion that *Myh9* is epistatic to *Myh10*, application of a myosin II-specific inhibitor, Blebbistatin, also markedly restored cilium formation in Myh10-knockdown cells (Fig. 2D). We noted that the cilia formed under these conditions were generally shorter than those in wild type control cells, which suggests incomplete rescue of Myh10 function by Myh9 inhibition. These data indicate that Myh9 activity is, at least partially, responsible for cilium inhibition caused by *Myh10* knockdown. We next asked whether Myh9 activity might function to inhibit ciliogenesis in the presence of high serum when ciliogenesis is typically low. As shown in Fig. 2E, knocking down *Myh9* expression indeed significantly increased ciliogenesis. These experiments indicate that *Myh9* suppresses ciliogenesis under non-permissive conditions (e.g. high concentration of serum) and *Myh10* can counteract this activity.

### *Myh10* Antagonizes *Myh9* and Increases Actin Dynamics

**Figure 3:**
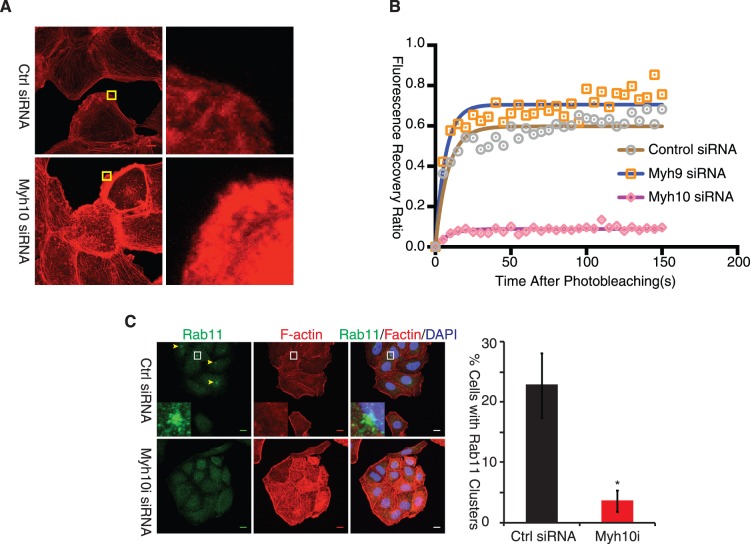
Myh10 Promotes Actin Network Dynamics. (A) Myh10 knockdown stabilized actin network. ARPE-19 cells were transfected with control and Myh10 siRNA and stained with phalloidin-555 to label F-actin. Yellow insets were magnified to allow better visualization of F-actin on the cell edge. Scale bars: 5 µm. (B) Myh10 knockdown inhibited actin network dynamics. ARPE-19 cells were transfected with GFP-actin construct first and then with control, Myh9 or Myh10 siRNA. GFP positive cells were then subjected to FRAP analysis. FRAP data from 5 individual cells were averaged, plotted, and superimposed with fitted dissociation curve. Myh9 siRNA: organge squares and blue solid line; Myh10 siRNA: gray circles and brown solid line; Control siRNA: pink diamonds and magenta solid line. (C) Myh10 knockdown reduced pericentrosomal recycling endosome clusters. ARPE-19 cells were transfected with control (upper panel) and Myh10 (lower panel) siRNA and fixed with 4% PFA 48 hours after transfection. Recycling endosomes (yellow arrowheads) were labeled with mouse anti-Rab11 antibody (green). Cells were also labeled with phalloidin-555 to show boundaries. Percentage of cells with perinuclear Rab11 clusters was quantified and plotted (right panel). Scale bars: 10 µm. *, t-test p<0.01.

*Myh9* is known to stabilize actin bundles and the filament network [18]. We therefore asked whether *Myh10* might oppose *Myh9* and increase actin network dynamics. To test this possibility, we first performed Phalloidin staining in ARPE-19 cells (an immortalized human retina pigment epithelia cell line) to examine the influence of *Myh10* knockdown on the F-actin network. As reported previously, *Myh10* knockdown did not disrupt stress fiber formation (S2A Figure, arrow heads), which is controlled by *Myh9* [18]. *Myh10* knockdown, however, dramatically increased F-actin staining intensity at the cell edge and the cell cortex (Fig. 3A and S2B Figure), suggesting the stabilization of the F-actin network. To quantitatively measure actin dynamics, we performed a fluorescence recovery after photobleaching (FRAP) assay using β-actin-GFP in ARPE-19 cells. We found that in contrast to *Myh9* knockdown, which accelerated the initial phase of fluorescence recovery after photobleaching, *Myh10* knockdown dramatically blocked the recovery (Fig. 3B, S2C Figure). This result indicates that loss of *Myh10* leads to a more stable actin network.

The areas with enhanced F-actin staining in *Myh10*-knockdown cells were also enriched for p34/Arc, a marker for the branched actin network (S2D Figure). Branched actin network has been proposed to prevent the concentration of pericentrosomal recycling endosomes that provide membranes for primary cilium growth [7], [17]. We therefore examined whether Myh10 regulates pericentrosomal recycling endosome cluster formation. As shown in Fig. 3C, Myh10 knockdown dramatically reduced the percentage of cells with Rab11-positive recycling endosomes clusters, consistent with the function of Myh10 in maintaining actin dynamics. Taken together, *Myh10*, contrary to *Myh9*, functions to maintain a dynamic actin network and supports pericentrosomal recycling endosome cluster formation.

### *Myh10*-Dependent Actin Dynamics Regulates Pericentrosomal *Pcm-1/Cep290* Localization

**Figure 4:**
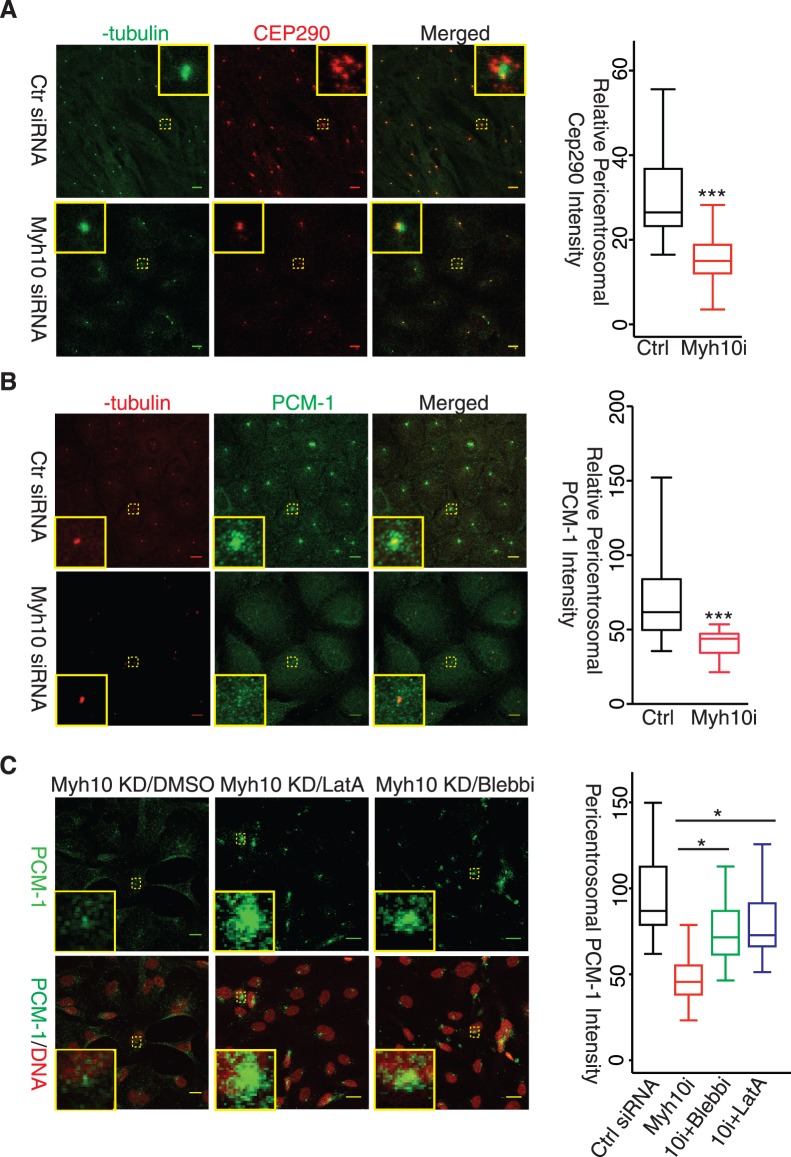
Myh10 Regulates Cep290-Pcm-1 Centriolar Satellites Organization. (A) Myh10 knockdown prevented Cep290 centriolar satellites formation. RPE-Mchr1^GFP^ cells were transfected with control (left panel, upper row) and Myh10 (left panel, lower row) siRNA and immunostained with γ-tubulin (left panel, green) and Cep290 (left panel, red) antibody. Yellow insets were magnified to allow better visualization of representative images. A 30-pixel diameter circle around the centrosome was drawn for each cell and Cep290 staining intensity within that circle was quantified and plotted (right panel). Scale bars: 10 µm. t-test, p<0.001. (B) Myh10 knockdown reduced Cep290 staining in the pericentrosomal region. Control and Myh10 siRNA transfected RPE-Mchr1^GFP^ cells were fixed and stained for γ-tubulin (red) and PCM-1 (green) (left panel). Pericentrosomal PCM-1 staining was quantified and plotted as described in (A). Yellow insets were magnified to allow better visualization of representative images. Scale bars: 10 µm. t-test, p<0.001. (C) Actin destabilization and Myh9 inhibition restored centriolar satellite PCM-1. Myh10 knockdown RPE-Mchr1^GFP^ cells were treated with DMSO (left panel), 20 nM latrunculin A (middle) and 25 µM blebbistatin for 24 hours then labeled with PCM-1 antibody (green). Yellow boxes show magnified representative images of pericentrosomal PCM-1 cluster. Pericentrosomal PCM-1 cluster intensity was quantified and plotted (right panel). Black, control siRNA with DMSO; red, Myh10 siRNA with DMSO; green, Myh10 siRNA with 25 µM blebbistatin; purple, Myh10 siRNA with 20 nM latrunculin A. *, t-test p<0.01.

The requirement of *Myh10* for the assembly of pericentrosomal recycling endosome pool prompted us to examine whether *Myh10*-dependent actin dynamics are also necessary for clustering other pericentrosomal components required for cilium formation. We examined Cep290 and PCM-1 proteins, which are co-localized to centriolar satellites and recruit Rab8 to facilitate the fusion of preciliary vesicles to the ciliary membrane [8]. In *Myh10*-knockdown cells pericentrosomal Cep290 staining showed a marked decrease in signal intensity (Fig. 4A) whereas *Myh9* knockdown had little effect (S3A Figure). More detailed examination revealed a specific loss of Cep290 at the centriolar satellite whereas centrosome-associated Cep290 was retained (Fig. 4A, left panels, insets). PCM-1 was also dispersed from the pericentrosomal region in *Myh10*-knockdown cells (Fig. 4B, and S3B Figure). The overall protein levels of *Cep290* and *PCM-1* remained unaltered in *Myh10*-knockdown cells (S3C Figure). These results show that the *Cep290-PCM-1* preciliary complex is mis-localized in *Myh10*-deficient cells.

Next, we tested if actin dynamics underlie *Myh10*-dependent pericentrosomal structure organization. We treated *Myh10*-knockdown RPE-Mchr1^GFP^ line with a low dosage (20 nM) of Latrunculin A, an actin depolymerization reagent, to increase actin dynamics in Myh10-knockdown cells. 24 hours after Latrunculin A (LatA) treatment, pericentrosomal *PCM-1* was partially restored (Fig. 4C). Inhibiting *Myh9* by the myosin II-specific inhibitor, Blebbistatin (25 µM), also significantly restored *PCM-1* clusters in *Myh10* knockdown cells (Fig. 4C, Blebbi panels). These data indicate that *Myh10* promotes pericentrosomal concentration of the *Cep290-PCM-1* complexes by opposing *Myh9* and increasing dynamics of the actin cytoskeleton.

### *Myh10* Expression Is Upregulated during Ciliogenesis by Tubulin Acetyltransferase *Mec-17*

**Figure 5:**
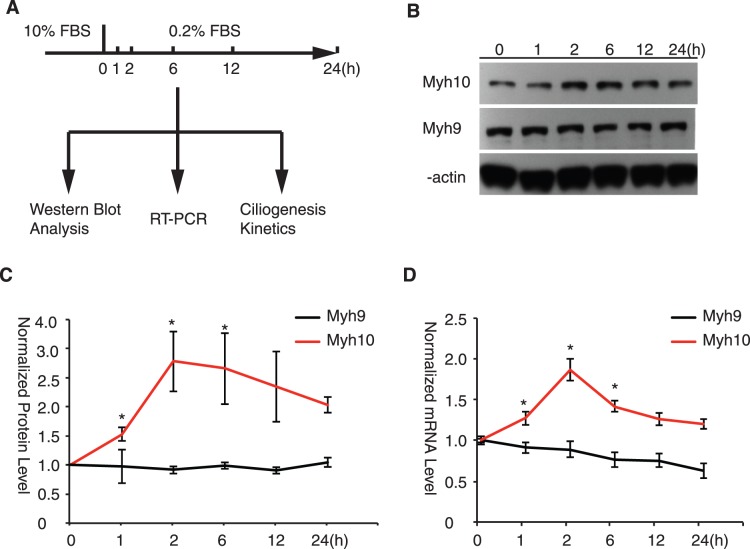
Myh10 Is Upregulated During Ciliogenesis. (A) Schematic illustration of selected gene expression analysis during ciliogenesis in time course. (B) Myh10 protein level is upregulated during ciliogenesis. RPE-Mchr1^GFP^ cells serum starved (0.2% FBS) for 0, 1, 2, 6, 12 and 24 hours were collected for western blot analysis of Myh10 (upper panel) and Myh9 (middle panel) protein level. β-actin was used an internal loading control. (C) Relative protein bands from (B) were quantified using Fiji gel analysis function. Results from three independent experiments were quantified, normalized to β-actin loading control and averaged. *, t-test p<0.01. (D) Myh10 mRNA is upregulated during ciliogenesis. mRNA samples collected at indicated serum starvation time points were used as template for real-time PCR to measure Myh9 (black line) and Myh10 (red line) mRNA expression levels. Error bars represent standard deviations (s.d.) from triple biological replicates.

The fact that Myh10 promotes cilium elongation prompted us to examine whether *Myh10* expression is regulated during cilium formation. We monitored the expression of *Myh10* during cilium formation induced by serum starvation (Fig. 5A). Interestingly, *Myh10* protein levels gradually increased during the early phase of cilium formation (0–12 h after starvation, Fig. 5B, C). In contrast, *Myh9* protein levels were relatively constant. Real-time PCR analysis showed that *Myh10* mRNA levels increased within 2 hours of serum starvation whereas *Myh9* mRNA level was not induced (Fig. 5D). These data reveal that relative abundance of *Myh10* vs. *Mhy9* increases in response to serum starvation, a condition that would favor primary cilia formation.

**Figure 6:**
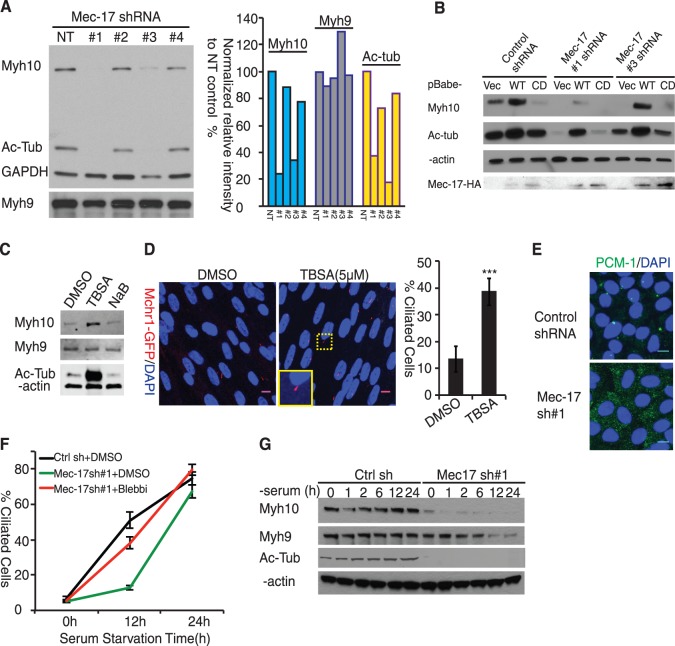
Mec-17 Controls Myh10 Expression to Regulate Ciliogenesis Kinetics. (A) Mec-17 knockdown reduced Myh10 protein expression. RPE-Mchr1^GFP^ cells were transduced with 4 different shRNA lentiviruses targeting Mec-17. Knockdown efficiency was assessed by acetylated tubulin level. Myh10 and Myh9 protein level was analyzed by western blotting. GAPDH was used as an internal loading control (left panal). Intensity of protein bands in left panel were quantified using FIJI gel analysis tools and normalized to GAPDH level. Relative bands intensity of each protein were further normalized to the NT control and plotted (right panel). (B) Myh10 protein expression depends on Mec-17 catalytic activity. Control, #1 and #3 Mec-17 knockdown ARPE-19 cells were infected with pBabe retrovirus expressing HA-tagged Mec-17 wild type (WT) or Mec-17 catalytic dead mutant (CD). Myh10 protein expression was examined by western blot. Acetylated tubulin was used as a positive control to indicate the presence of Mec-17 catalytic activity. β-actin was used as an internal protein loading control. HA blot showed expression of Mec-17-HA fusion protein. (C) Inhibiting tubulin deacetylase activity upregulates Myh10 expression. RPE-Mchr1^GFP^ cells were treated with different deacetylase inhibitors, lane 1: DMSO; lane 2: 5 µM tubastatin (TBSA); lane 3: 5 mM sodium butyrate(NaB) and analyzed for Myh9 and Myh10 expression. β-actin was used as an internal loading control. (D) Inhibiting HDAC6 activity enhanced spontaneous ciliogenesis. RPE-Mchr1^GFP^ cells cultured in DMEM/20% FBS were switched to 5% FBS containing DMSO or 5 µM tubastatin A for 24 hours. Cells were fixed by methanol and visualized under a confocal microscopy. Mchr1-GFP (pseudo-colored red) was used to identify and quantify cilia. Yellow inset shows magnified image of representative cilium. Results from 3 biological replicates were averaged and plotted (right panel). DAPI (blue) was used to label the nuclei. ***, t-test p<0.001. Scale bar: 10 µm. (E) Mec-17 is required for centrosomal PCM-1 cluster organization. Mec-17 #1 shRNA transduced cells were methanol fixed and stained with rabbit PCM-1 antibody to examine the intracelluar distribution of PCM-1 proteins (lower panel, green). (F) Blebbistatin accelerated ciliogenesis in Mec-17 KD cells. Non-target control (NT, black line) and Mec-17 #1 KD cells were treated with DMSO (green line) or 25 µM blebbistatin (red line) in DMEM with 0.2% FBS and quantified for percent of ciliated cells at 0, 12 and 24 hours. Error bars represent standard deviation from triple biological replicates. (G) Reduced expression of Myh9 during late stage of ciliogenesis compensates for Myh10 loss to promote cilium formation in Mec-17-knockdown cells. Non-target control and Mec-17 KD #1 cells were serum starved and collected at indicated time points to monitor Myh9 and Myh10 expression. Acetylated tubulin was used to show the efficient knockdown of Mec-17. β-actin was used an internal protein loading control.

We next investigated the mechanism by which *Myh10* is induced prior to cilium formation. As cilia are heavily enriched for acetylated microtubules, we determined if microtubule acetyltransferase *Mec-17* affects *Myh10* expression. We transduced RPE-Mchr1^GFP^ cells with several independent *Mec-17* shRNA lentiviral particles. Two (#1 and #3) independent shRNA constructs effectively reduced acetylated tubulin levels (Fig. 6A) and *Mec-17* mRNA (S4A Figure). Remarkably, in both *Mec-17* shRNA knockdown cell lines, *Myh10* protein level was markedly reduced (Fig. 6A) in the presence of serum whereas Myh9 remained relatively unchanged. The decrease in *Myh10* protein abundance was not due to protein degradation as inhibiting proteasome function by MG132 did not restore Myh10 level (data not shown). Indeed, quantitative PCR detected a significant reduction of *Myh10* mRNA (∼60–70%) in *Mec-17*-knockdown cells (S4B Figure), suggesting that *Mec-17* regulates *Myh10* protein expression via a transcriptional mechanism. A modest reduction of *Myh9* mRNA was also observed although its protein level was not affected (S4B Figure). Importantly, *Myh10* was not induced by serum starvation in *Mec-17* knockdown cells (Fig. 6G, Top Panel). Collectively, these results showed that *Mec-17* is required for *Myh10* upregulation in response to serum starvation.

The tubulin acetyltransferase *Mec-17* has been indicated to possess both catalytic-dependent and –independent functions [23]. To determine if *Myh10* expression depends on *Mec-17* catalytic activity, we expressed wild type and catalytic-dead (CD) D157N mutant of mouse *Mec-17* [24]. As shown in Fig. 6B, wild type *Mec17* restored *Myh10* expression to different degrees in two *Mec-17* KD lines that correlated with the extent of tublin acetylation whereas catalytic-dead (CD) D157N mutant was unable to restore tubulin acetylation or *Myh10* expression. Of interest, over-expression of *Mec-17* in control KD cells increased tublin acetylation and *Myh10* levels (Fig. 6B, lane Control shRNA, WT). These results indicate that *Mec-17* induces *Myh10* via its acetyltransferase activity, likely by acetylating microtubules. Indeed, treatment with tubastatin A (TBSA), a specific inhibitor for tubulin deacetylase HDAC6 [25] significantly increased tubulin acetylation and *Myh10* expression in RPE-Mchr1^GFP^ cells, whereas sodium butyrate (NaB), a HDAC inhibitor that does not target HDAC6, had no effect (Fig. 6C). Importantly, TBSA treatment significantly increased spontaneously formed cilia in the presence of serum, which further supports the critical role of *Myh10* protein expression in cilium formation (Fig. 6D). Altogether, these experiments support the conclusion that *Mec-17* promotes Myh10 expression by increasing microtubule acetylation.

If *Mec-17* is required for *Myh10* expression, one would expect defective ciliogenesis in *Mec-17* knockdown cells. Indeed, we found that *Mec-17* knockdown caused PCM-1 dispersion (Fig. 6E) and ciliogenesis was significantly reduced in the early phase (Fig. 6F, 12 hours after serum withdrawal). Similar to *Myh10* knockdown cells, the ciliogenesis defect in *Mec-17* KD cells can be corrected by Blebbistatin treatment that inactivates Myh9 (Fig. 6F, red line at 12 hours). Intriguingly, cilium formation in *Mec-17* knockdown cells eventually recovered at later time points (Fig. 6F, green), as it was previously reported [12]. This later phase recovery of ciliogenesis is also consistent with normal cilia formation observed in *Mec-17*-deficient mice [14], [15]. Analysis of *Myh9* protein in *Mec-17*-knockdown cells revealed that it was decreased in later time points after serum starvation (12–24 hours, Fig. 6G). Given the inhibitory activity of Myh9 on cilium formation, the loss of Myh9 could explain the eventual recovery of cilium formation in *Mec-17* knockdown cells. Our data reveal that *Mec17* regulates ciliogenesis, at least in part, through Myh10 induction.

## Discussion

The formation of primary cilium requires polarized growth of the membrane material delivered by the PPC complex and a buildup of acetylated microtubules. Our study suggests that these processes are coordinated by myosin II and tubulin acetyltransferase *Mec-17*. Actin cytoskeleton, especially branched actin network, plays an inhibitory role in PPC complex recruitment. Evidence suggests that the disassembly of the actin network is required for cilium formation [7], [17]. In this study, we have identified non-muscle myosin IIA (*Myh9*) and IIB (*Myh10*) as the key regulators of the actin cytoskeleton that controls PPC complex and cilium formation. Pericentrosomal recycling endosomes are one of the main organelles that provide a membrane source to support cilia growth [26]. Cep290-PCM-1 complex is also localized to the pericentrosomal region where it recruits Rab8 to facilitate the vesicular trafficking to the cilia [8]. Our results show that Myh10 is required for pericentrosomal recruitment of Cep290, PCM-1 as well as Rab11-positive recycling endosomes. Importantly, defects in PPC recruitment in *Myh10* KD cells can be partially rescued by inhibiting Myh9 or destablizing actin cytoskeleton (Fig. 4). Thus, *Myh9* and *Myh10* are the critical regulators of the actin cytoskeleton that controls the concentration of cilia-associated proteins and organelles for ciliogenesis. It is also important to note that *Myh10* is required to maintain pericentrosomal organization of recycling endosome clusters and Cep290-PCM-1 satellites even in the presence of serum. This finding suggests that basal level of Myh10 and actin dynamics may be a pre-requisite to prime ciliogenesis by facilitating preciliary structure assembly.

Despite their homology, *Myh9* and *Myh10* knockdown display an opposite phenotype in ciliogenesis. Myosin IIA (Myh9) has been shown to form oligomers to stablize and promote the assembly of F-actin-based structures [18], [27]–[29]. Our analysis indicates that Myh10 opposes Myh9 activity, potentially by forming hetero-oligomers (Fig. 2F), to increase actin dynamics and stimulate cilium formation (Fig. 3). The antagonistic activity of Myh9 and Myh10 suggests that the relative abundance or activity of Myh9 and Myh10 could determine the state of cilia. Therefore, the observed induction of *Myh10* upon serum starvation could potentially act as a part of the molecular switch that releases the break on cilogenesis enforced by the inhibitory actin cytoskeleton when accumulated Myh10 overcomes Myh9.

Interestingly, we found that *Myh10* expression is positively regulated by the tubulin acetyltransferase *Mec-17*. The regulation of *Myh10* requires *Mec-17* acetyltransferase activity (Fig. 6B), suggesting that microtubule acetylation is coupled to *Myh10* transcriptional induction. Indeed, an increase in tubulin acetylation by inhibiting HDAC6 also led to *Myh10* induction and enhanced cilium formation (Fig. 6C–D). As cilia primarily consist of acetylated microtubules, this finding suggests an intriguing link between microtubule acetylation and the activation of cilium assembly. Indeed, knocking down *Mec-17* prevented *Myh10* induction and delayed normal ciliogenesis in quiescent cells (Fig. 6F). This microtubule acetylation-dependent regulation of gene transcription may represent a novel function of microtubule network to regulate cellular signaling events. We recently reported similar regulation in anti-inflammatory response of LPS-treated macrophage cells and suggested that this regulation may present in broader cellular processes. In macrophage anti-inflammatory response, LPS-treatment stimulated tubulin acetylation which amplified the p38 kinase cascade and activated downstream SP1-dependent transcription of IL-10 [30]. We hypothesized that similar acetylated microtubule-dependent signal amplification mechanisms may be involved in the induction of Myh10 during cell quiescence-induced ciliogenesis but may depend on other kinases and transcription factors. Although further experiments would be required to determine how acetylated microtubules control *Myh10* expression, the *Mec-17*-dependent *Myh10* induction provides a potential mechanism for a coordinated regulation of acetylation of microtubules and delivery of ciliary membranes and proteins, two key elements for cilium assembly. Interestingly, *Mec-17* mRNA level is also upregulated by serum starvation (S4C Figure). These findings suggest a simple model for ciliogenesis where *Mec17*, upon cellular quiescence, is activated to catalyze microtubule acetylation for building ciliary axoneme. Microtubule acetylation also induces *Myh10* expression, which increases the dynamics of the actin network by counteracting Myh9, thereby assisting the assembly of PPC and enabling delivery of membrane and protein components for cilium growth. Recent genetic mouse models suggest that *Mec-17* is dispensable for *in vivo* ciliogenesis [14], [15], our data, however, indicates that *Mec-17* regulates in the early steps of cilia formation and kinetics control. Whether a faster kinetics of ciliogenesis is physiological relevant in any *in vivo* context remains to be investigated. Nevertheless, our finding that increasing tubulin acetylation facilitates ciliogenesis may point to a potential intervention of various human diseases resulting from cilia defects and therefore may be worth further testing in animal models of ciliopathies.

## Materials and Methods

### Cell Lines, Constructs, Sirnas, Antibodies and Chemical Reagents

Three cell lines were used in this study: RPE-Mchr1^GFP^ cells were a kind gift from Dr. Peter Jackson’s laboratory at Genentech, Inc. IMCD3 cells were obtained from Dr. Nicholas Katsanis laboratory at Duke University Medical Center. ARPE-19 cells (ATCC #CRL-2302) were purchased from Duke University Cell Culture Facility. All cells were maintained and passaged according to provider’s instructions. Myh9-GFP (Addgene plasmid 11347) and Myh10-GFP (Addgene plasmid 11348) constructs were purchased from addgene and were originally constructed by Dr. Robert S. Adelstein’s laboratory. GFP-actin was obtained from Dr. Michael D. Ehlers’ laboratory. MSCV-Puro retroviral vector was a kind gift from Dr. Xiao-fan Wang’s lab at Duke University. Human Myh10 cDNA sequence was amplified by PCR reactions (primers were listed as table 1) and cloned into a modified MSCV-puro vector to generate MSCV-HA-Flag-Myh10. Human pLKO.1 Mec-17 shRNA constructs were purchased from Sigma (#1 shRNA: TRCN0000263600; #3 shRNA: TRCN0000263597). pLKO.1 non-target control constructs was also a gift from Dr. Xiao-fan Wang’s lab at Duke University. Antibodies used in this study includes: rabbit anti-glu-tubulin (Millipore #AB3201, 1∶200), mouse anti-acetylated-tubulin (sigma #T7451, 1∶1000 for IF and 1∶3000 for WB), mouse anti-γ-tubulin (sigma #T5326, 1∶2000), rabbit anti-γ-tubulin (sigma #T3559, 1∶500), mouse anti-Rab11 (BD Transduction Laboratories #610656, 1∶200), rabbit anti-Cep290 (Bethyl Laboratories, Inc. #IHC-00365, 1∶300), rabbit anti-PCM-1 (Epitomics #T2443, 1∶200), mouse anti-Myh10 (Developmental Studies Hybridoma Bank #CMII 23, 1∶1000 for western blot), rabbit anti-Myh9 (Santa Cruz #sc-98978, 1∶1000 for western blot). siRNA duplex sequences used in this study are listed in table 2. Chemical reagents used in this study includes: Blebbistatin (Sigma #B0560), Latrunculin A (Sigma #L5163), Tubastatin A (BioVision #1724-1), sodium butyrate (Sigma #B5887).

**Table 1:** Primer sequences for cloning and real-time PCR.

Cloning	Myh10 (Hs.)	Forward	5′ATGGCGCAGAGAACTGGACT
		Reverse	5′TTACTCTGACTGGGGTGGCTG
Real-time PCR	Gli1 (Hs.)	Forward	5′GGGTGCCGGAAGTCATACTC
		Reverse	5′GCTAGGATCTGTATAGCGTTTGG
	Myh9 (Hs.)	Forward	5′CAGCAAGCTGCCGATAAGTAT
		Reverse	5′CTTGTCGGAAGGCACCCAT
	Myh10 (Hs.)	Forward	5′TGGTTTTGAGGCAGCTAGTATCA
		Reverse	5′AGTCCTGAATAGTAGCGATCCTT
	Mec-17 (Hs.)	Forward	5′GGCCCAGAATCTTTCCGCTC
		Reverse	5′GATGCAAAGTGGTTCTACCTCAT
	Myh9 (Mm.)	Forward	5′GGCCCTGCTAGATGAGGAGT
		Reverse	5′CTTGGGCTTCTGGAACTTGG
	Myh10 (Mm.)	Forward	5′GGAATCCTTTGGAAATGCGAAGA
		Reverse	5′GCCCCAACAATATAGCCAGTTAC

**Table 2:** siRNA sequences used in this study.

MYH10 (Hs.) #1	UUCUGUACAAGUUUGGGUCCAAUUC	Invitrogen
MYH10 (Hs.) #1	GCAAUACAGUGGGACAGUU[dT][dT]	Sigma
MYH9 (Hs.)	GAGUCUGAGCGUGCUUCCAGGAAUA	Invitrogen

### Cell Culture, Transfection and Cilia Formation Assay

RPE-Mchr1^GFP^ cells were maintained in DMEM (Gibco) containing 10% FBS (Fetal Bovine Serum) in a 37°C humidified incubator with 5% CO_2_. ARPE-19 and IMCD3 cells were maintained in DMEM:F12 medium (Gibco) containing 10% FBS. Cells were passaged at 80%–90% confluence at a regular basis. To induce cilia formation, cells were allow to grow confluent on a coverslip before switching to DMEM or DMEM: F12 containing 0.2% FBS. For the time course study of ciliogenesis, confluent cell layers were first stimulated with 20% FBS for 6 hours to disassemble spontaneously formed cilia and then switched to medium containing 0.2% FBS. Transient transfections of plasmids or siRNA duplexes were performed with lipofectamine 2000 or RNAiMAX (Life Technologies) following manufacturer’s guide. For stable transfection using lentivirus or retrovirus, virus was packaged with appropriate packaging plasmids (Δ8.9 and pCMV-VSVG for lentivirus; pCL-Ampho for retrovirus) in 293T cells. Viruses were collected 48 hours after transfection, filtered through 0.45 µm sterile cellulose acetate membrane filters. Viruses were added to cell culture medium and selected with puromycin (1 µg/ml) for a week. Survived cells were then collected or passaged for analysis. Multiple rounds of infection were performed in a need basis. For non-virus based stable transfection, plasmids were delivered into the cell by Lipofectamine 2000 transfection. Transfected cells were then split at 1∶10 and selected with corresponding antibiotics (1 µg/ml puromycin or 0.5 mg/ml G418) for 3 weeks. Individual cell colonies were picked and expanded for further analysis. To maintain all stable cell lines, culture medium contained antibiotics (1 µg/ml puromycin or 0.5 mg/ml G418).

### Immunofluorescence and F-Actin Staining

For immunofluorescence, cells were fixated with ice-cold methanol (for antibodies targeting acetylated-tubulin, γ-tubulin, Cep290 and PCM-1) or 4% paraformaldehyde in PBS (for Rab11), washed with PBS, blocked with 10% normal goat serum, then incubated with primary antibodies diluted in 10% normal goat serum overnight in 4°C. Primary antibodies were rinsed with PBS and incubated with secondary antibody in 10% normal goat serum for 1 hour and then rinsed and mounted on glass slides for microscopy imaging. For F-actin staining, cells were fixated in pre-warmed (37°C) 4% paraformaldehyde and rinsed with PBS, then incubated with phalloidin-555 (Cytoskeleton #PHDH1-A) for 1 hour. Coverslips were then mounted on glass slides for imaging on a Zeiss 780 microscopy.

### Protein Interaction

To detect protein-protein interaction between Myh9 and Myh10, different amount of MSCV-HA-Flag-Myh10 plasmids (0, 1.5, 3.0, 6.0, 9.0 ug) were transfected into 293T cells using lipofectamine 2000 (Invitrogen) following the manufacturer’s guide. Cells were collected 48 hours after transfection and lysed in immunoprecipitation buffer (150 mM NaCl, 20 mM pH 8.0 Tris.HCl, 0.5 mM EDTA, 0.5% NP-40). Lysate containing 1 mg total protein were incubated with anti-FLAG M2 affinity gel (Sigma #A2220) for 2 hours at 4°C and washed 3 times with same lysis buffer. Precipitates were then mixed with protein loading buffer and boiled for western blot analysis.

### Fluorescence Recovery after Photobleaching (Frap)

ARPE-19 cells were first transfected with Actin-GFP plasmid and then with control, Myh9 and Myh10 siRNA. 48 hours after siRNA transfection, cells were switched to warm phenol-free DMEM for FRAP analysis. Cells were imaged for 10 seconds, then photobleached at selected regions and imaged for 3 minutes to allow fluorescence recovery. For each knockdown group, a total of five cells were used for analysis. Intensity data from 5 cells were analyzed using FRAP profiler plugin for imageJ, averaged and plotted. The fluorescence recovery curve fitting was performed with Prism 6 software (GraphPad Software) under the exponential association function.
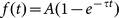



### Real-Time Pcr Analysis of Gene Expression

Total RNA was extracted from cells using RNAeasy mini kit (Qiagen), quantified using a spectrometer. 1 ug of total RNA from each sample was reverse transcribed using Promega M-MLV Reverse Transcriptase to be used as template for realtime PCR analysis. cDNA and primers for specific genes (listed in table 1) were mixed in realtime PCR master mix (Qiagen). Real-time PCR reaction was performed on a Realplex Mastercycler (Eppendorf). Data was analyzed with Excel.

### Data Processing and Statistical Analysis

All the quantifications were from three independent experiments. To quantify the percentage of ciliated cells, 100–150 randomly chosen cells were counted under the microscopy. Only cells with acetylated tubulin staining longer than 1 µm were considered as bearing mature cilia and counted as positive. For cilia length analysis, cilia lengths were determined using the line scan function in Fiji imaging processing software. Lengths of cilia were plotted as cumulative probability distribution curve. Data are demonstrated as mean ± s.d. Two-group hypothesis testing were performed with student t-test. Results were considered statistically significant when p<0.01.

## Supporting Information

S1 Figure **Myh10 is required for ciliogenesis.** (A) Efficient knockdown of Myh9 and Myh10 by siRNA in RPE-Mchr1^GFP^. Control, Myh10 #1, #2 and Myh9 siRNA duplexes were transfected in RPE-Mchr1^GFP^ cells. Cells were collected 48 hours after transfection for western blot analysis of Myh9 and Myh10 protein expression. (B) Efficient knockdown of Myh9 and Myh10 in IMCD3 cells. IMCD3 cells were transfected with mouse Myh9 and Myh10 siRNA duplexes. Cells were collected 48 hours after transfection for Q-PCR analysis of Myh9 (black) and Myh10 (red) transcripts expression.
(TIF)

S2 Figure **Myh10 knockdown stabilized F-actin network**. (A) Myh10 knockdown stabilized F-actin network and did not disrupt stress fibers. Phalloidin-555 staining of F-actin network of control and Myh10 knockdown ARPE-19 cells. Yellow insets were magnified to allow better visualization of stress fibers (arrowheads). (B) Myh10 knockdown enhanced cortex actin network. Myh10 knockdown APRE-19 cells were stained with phalloidin-555 to label the actin network and imaged under a confocal microscope. Optical sections (0.3 µm) from cell cortex to cell bottom were shown in sequence. All images are shown as gray scale pictures. (C) Myh10 and Myh9 displayed opposite activity on actin dynamics. GFP-actin expressing (transient) ARPE-19 cells were transfected with control, Myh9 and Myh10 siRNA duplexes and subjected for FRAP analysis. Cells were photobleached near the cell edge and allowed to recover for 3 minutes during which images were acquired and selected time points were shown as spectrum images. Black insets indicates photobleached regions. Scale bar: 5 µm. (D) Myh10 knockdown enhanced branched actin network on the cell periphery. Myh9 and Myh10 knockdown RPE-Mchr1^GFP^ cells were stained with rabbit anti-p34/Arc antibody to label branched actin network. Images were presented as spectrum. Red arrowheads indicate enhanced p34/Arc staining.
(TIF)

S3 Figure **Myh10 knockdown specifically affected centriolar satellite organization.** (A) Myh9 knockdown does not inhibit Cep290 centriolar satellite recruitment. Control and Myh9 knockdown RPE-Mchr1^GFP^ cells were stained with Cep290 (red) and γ-tubulin (green). White dash lines outline individual cell morphologies. Scale bar: 5 µm. (B) Myh10 knockdown caused PCM-1 dispersion from centriolar satellites. Myh10-knockdown cells were stained with PCM-1 (green) and γ-tubulin (red). Representative images of centrosomal regions are shown. Control KD: upper panel; Myh10 KD: lower panel. (C) Myh10 knockdown does not affect Cep290 and PCM-1 protein abundance. Control (left column) and Myh10 KD (right lane) RPE-Mchr1^GFP^ cells were collected for western blot analysis of Cep290 and PCM-1 protein levels.
(TIF)

S4 Figure ***Myh10*** **expression is regulated by** ***Mec-17*** **at the mRNA level during ciliogenesis.** Q-PCR quantification of *Mec-17* shRNA knockdown efficacy in ARPE-19 cells. ARPE-19 cells were infected with *Mec-17* shRNA lentivirus supernatant and selected with 1 ug/ml puromycin for a week. mRNA was extracted and reverse transcribed for Q- PCR analysis. *Mec-17* mRNA levels measured by Q-PCR were normalized to beta-actin control. The final results were presented as relative levels to non-target control shRNA-transduced cells. (A) *Mec-17* knockdown suppressed Myh10 mRNA expression. ARPE-19 cells transduced with #1 *Mec-17* shRNA lentivirus were harvested for Q-PCR analysis of Myh9 and Myh10 mRNA levels. Results are presented as relative levels to non-target control samples. (B) Mec-17 mRNA expression was up-regulated during ciliogenesis. RPE-Mchr1^GFP^ cells were harvested after different time points of serum starvation. mRNA samples from different time points were subjected to Q-PCR analysis of *Mec-17* mRNA levels. Results were presented as relative ratios to starting time point level.
(TIF)

## References

[1] Ansley SJ, Badano JL, Blacque OE, Hill J, Hoskins BE, et al (2003) Basal body dysfunction is a likely cause of pleiotropic Bardet-Biedl syndrome. Nature 425:628–633. 14520415 10.1038/nature02030

[2] Louie CM, Gleeson JG (2005) Genetic basis of Joubert syndrome and related disorders of cerebellar development. Hum Mol Genet 14 Spec No. 2:R235–242. 10.1093/hmg/ddi264 16244321

[3] Badano JL, Mitsuma N, Beales PL, Katsanis N (2006) The ciliopathies: an emerging class of human genetic disorders. Annu Rev Genomics Hum Genet 7:125–148. 16722803 10.1146/annurev.genom.7.080505.115610

[4] Garcia-Gonzalo FR, Reiter JF (2012) Scoring a backstage pass: mechanisms of ciliogenesis and ciliary access. J Cell Biol 197:697–709. 22689651 10.1083/jcb.201111146 PMC3373398

[5] Zaghloul NA, Katsanis N (2009) Mechanistic insights into Bardet-Biedl syndrome, a model ciliopathy. J Clin Invest 119:428–437. 19252258 10.1172/JCI37041 PMC2648685

[6] Westlake CJ, Baye LM, Nachury MV, Wright KJ, Ervin KE, et al (2011) Primary cilia membrane assembly is initiated by Rab11 and transport protein particle II (TRAPPII) complex-dependent trafficking of Rabin8 to the centrosome. Proc Natl Acad Sci U S A 108:2759–2764. 21273506 10.1073/pnas.1018823108 PMC3041065

[7] Kim J, Lee JE, Heynen-Genel S, Suyama E, Ono K, et al (2010) Functional genomic screen for modulators of ciliogenesis and cilium length. Nature 464:1048–1051. 20393563 10.1038/nature08895 PMC2929961

[8] Kim J, Krishnaswami SR, Gleeson JG (2008) CEP290 interacts with the centriolar satellite component PCM-1 and is required for Rab8 localization to the primary cilium. Hum Mol Genet 17:3796–3805. 18772192 10.1093/hmg/ddn277 PMC2722899

[9] Gerdes JM, Davis EE, Katsanis N (2009) The vertebrate primary cilium in development, homeostasis, and disease. Cell 137:32–45. 19345185 10.1016/j.cell.2009.03.023 PMC3016012

[10] L’Hernault SW, Rosenbaum JL (1985) Chlamydomonas alpha-tubulin is posttranslationally modified by acetylation on the epsilon-amino group of a lysine. Biochemistry 24:473–478. 3919761 10.1021/bi00323a034

[11] Akella JS, Wloga D, Kim J, Starostina NG, Lyons-Abbott S, et al (2010) MEC-17 is an alpha-tubulin acetyltransferase. Nature 467:218–222. 20829795 10.1038/nature09324 PMC2938957

[12] Shida T, Cueva JG, Xu Z, Goodman MB, Nachury MV (2010) The major alpha-tubulin K40 acetyltransferase alphaTAT1 promotes rapid ciliogenesis and efficient mechanosensation. Proc Natl Acad Sci U S A 107:21517–21522. 21068373 10.1073/pnas.1013728107 PMC3003046

[13] Hubbert C, Guardiola A, Shao R, Kawaguchi Y, Ito A, et al (2002) HDAC6 is a microtubule-associated deacetylase. Nature 417:455–458. 12024216 10.1038/417455a

[14] Kim GW, Li L, Gorbani M, You L, Yang XJ (2013) Mice lacking alpha-tubulin acetyltransferase 1 are viable but display alpha-tubulin acetylation deficiency and dentate gyrus distortion. J Biol Chem 288:20334–20350. 23720746 10.1074/jbc.M113.464792 PMC3711300

[15] Kalebic N, Sorrentino S, Perlas E, Bolasco G, Martinez C, et al (2013) alphaTAT1 is the major alpha-tubulin acetyltransferase in mice. Nat Commun 4:1962. 23748901 10.1038/ncomms2962

[16] Pugacheva EN, Jablonski SA, Hartman TR, Henske EP, Golemis EA (2007) HEF1-dependent Aurora A activation induces disassembly of the primary cilium. Cell 129:1351–1363. 17604723 10.1016/j.cell.2007.04.035 PMC2504417

[17] Cao J, Shen Y, Zhu L, Xu Y, Zhou Y, et al (2012) miR-129-3p controls cilia assembly by regulating CP110 and actin dynamics. Nat Cell Biol 14:697–706. 22684256 10.1038/ncb2512

[18] Even-Ram S, Doyle AD, Conti MA, Matsumoto K, Adelstein RS, et al (2007) Myosin IIA regulates cell motility and actomyosin-microtubule crosstalk. Nat Cell Biol 9:299–309. 17310241 10.1038/ncb1540

[19] Lecuit T, Lenne PF, Munro E (2011) Force generation, transmission, and integration during cell and tissue morphogenesis. Annu Rev Cell Dev Biol 27:157–184. 21740231 10.1146/annurev-cellbio-100109-104027

[20] Mukhopadhyay S, Wen X, Chih B, Nelson CD, Lane WS, et al (2010) TULP3 bridges the IFT-A complex and membrane phosphoinositides to promote trafficking of G protein-coupled receptors into primary cilia. Genes Dev 24:2180–2193. 20889716 10.1101/gad.1966210 PMC2947770

[21] Corbit KC, Aanstad P, Singla V, Norman AR, Stainier DY, et al (2005) Vertebrate Smoothened functions at the primary cilium. Nature 437:1018–1021. 16136078 10.1038/nature04117

[22] Wei Q, Adelstein RS (2000) Conditional expression of a truncated fragment of nonmuscle myosin II-A alters cell shape but not cytokinesis in HeLa cells. Mol Biol Cell 11:3617–3627. 11029059 10.1091/mbc.11.10.3617 PMC15019

[23] Topalidou I, Keller C, Kalebic N, Nguyen KC, Somhegyi H, et al (2012) Genetically separable functions of the MEC-17 tubulin acetyltransferase affect microtubule organization. Curr Biol 22:1057–1065. 22658602 10.1016/j.cub.2012.03.066 PMC3382010

[24] Friedmann DR, Aguilar A, Fan JY, Nachury MV, Marmorstein R (2012) Structure of the alpha-tubulin acetyltransferase, alpha TAT1, and implications for tubulin-specific acetylation. Proceedings of the National Academy of Sciences of the United States of America 109:19655–19660. 23071314 10.1073/pnas.1209357109 PMC3511727

[25] Butler KV, Kalin J, Brochier C, Vistoli G, Langley B, et al (2010) Rational Design and Simple Chemistry Yield a Superior, Neuroprotective HDAC6 Inhibitor, Tubastatin A. Journal of the American Chemical Society. 132:10842–10846. 10.1021/ja102758v PMC2916045 20614936

[26] Nachury MV, Seeley ES, Jin H (2010) Trafficking to the ciliary membrane: how to get across the periciliary diffusion barrier? Annu Rev Cell Dev Biol 26:59–87. 19575670 10.1146/annurev.cellbio.042308.113337 PMC2952038

[27] Liu X, Hong MS, Shu S, Yu S, Korn ED (2013) Regulation of the filament structure and assembly of Acanthamoeba myosin II by phosphorylation of serines in the heavy-chain nonhelical tailpiece. Proc Natl Acad Sci U S A 110:E33–40. 23248285 10.1073/pnas.1219727110 PMC3538215

[28] Smutny M, Cox HL, Leerberg JM, Kovacs EM, Conti MA, et al (2010) Myosin II isoforms identify distinct functional modules that support integrity of the epithelial zonula adherens. Nat Cell Biol 12:696–702. 20543839 10.1038/ncb2072 PMC3428211

[29] Murakami N, Kotula L, Hwang YW (2000) Two distinct mechanisms for regulation of nonmuscle myosin assembly via the heavy chain: phosphorylation for MIIB and mts 1 binding for MIIA. Biochemistry 39:11441–11451. 10985790 10.1021/bi000347e

[30] Wang B, Rao YH, Inoue M, Hao R, Lai CH, et al (2014) Microtubule acetylation amplifies p38 kinase signalling and anti-inflammatory IL-10 production. Nat Commun 5:3479. 24632940 10.1038/ncomms4479 PMC4000527

