# Supplementary figures and images for: A Mec17-Myosin II Effector Axis Coordinates Microtubule Acetylation and Actin Dynamics to Control Primary Cilium Biogenesis
Source: PLoS One. 2014 Dec 10;9(12):e114087. doi: 10.1371/journal.pone.0114087 (PMC4262394; doi:10.1371/journal.pone.0114087)

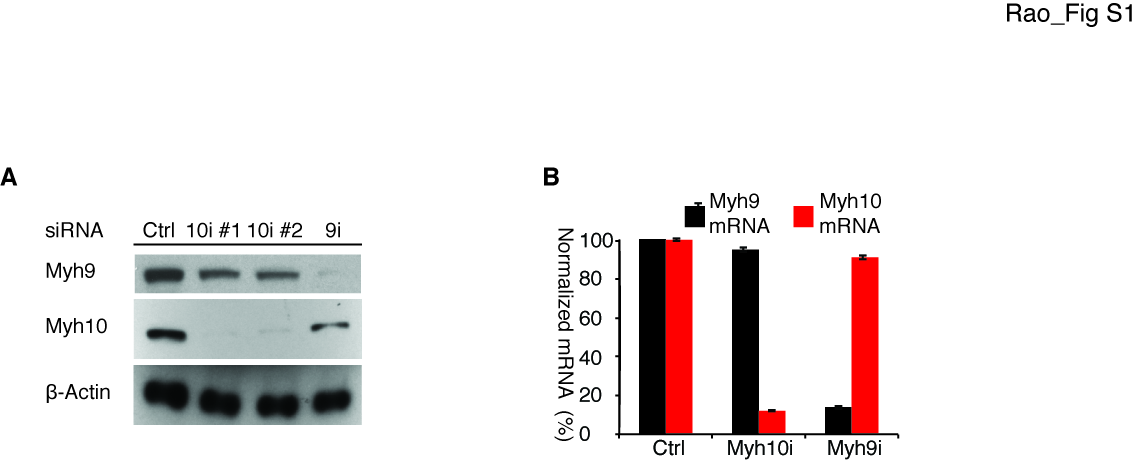

Supplement: S1 Figure — Myh10 is required for ciliogenesis. (A) Efficient knockdown of Myh9 and Myh10 by siRNA in RPE-Mchr1GFP. Control, Myh10 #1, #2 and Myh9 siRNA duplexes were transfected in RPE-Mchr1GFP cells. Cells were collected 48 hours after transfection for western blot analysis of Myh9 and Myh10 protein expression. (B) Efficient knockdown of Myh9 and Myh10 in IMCD3 cells. IMCD3 cells were transfected with mouse Myh9 and Myh10 siRNA duplexes. Cells were collected 48 hours after transfection for Q-PCR analysis of Myh9 (black) and Myh10 (red) transcripts expression. (TIF) [file pone.0114087.s001.tif]

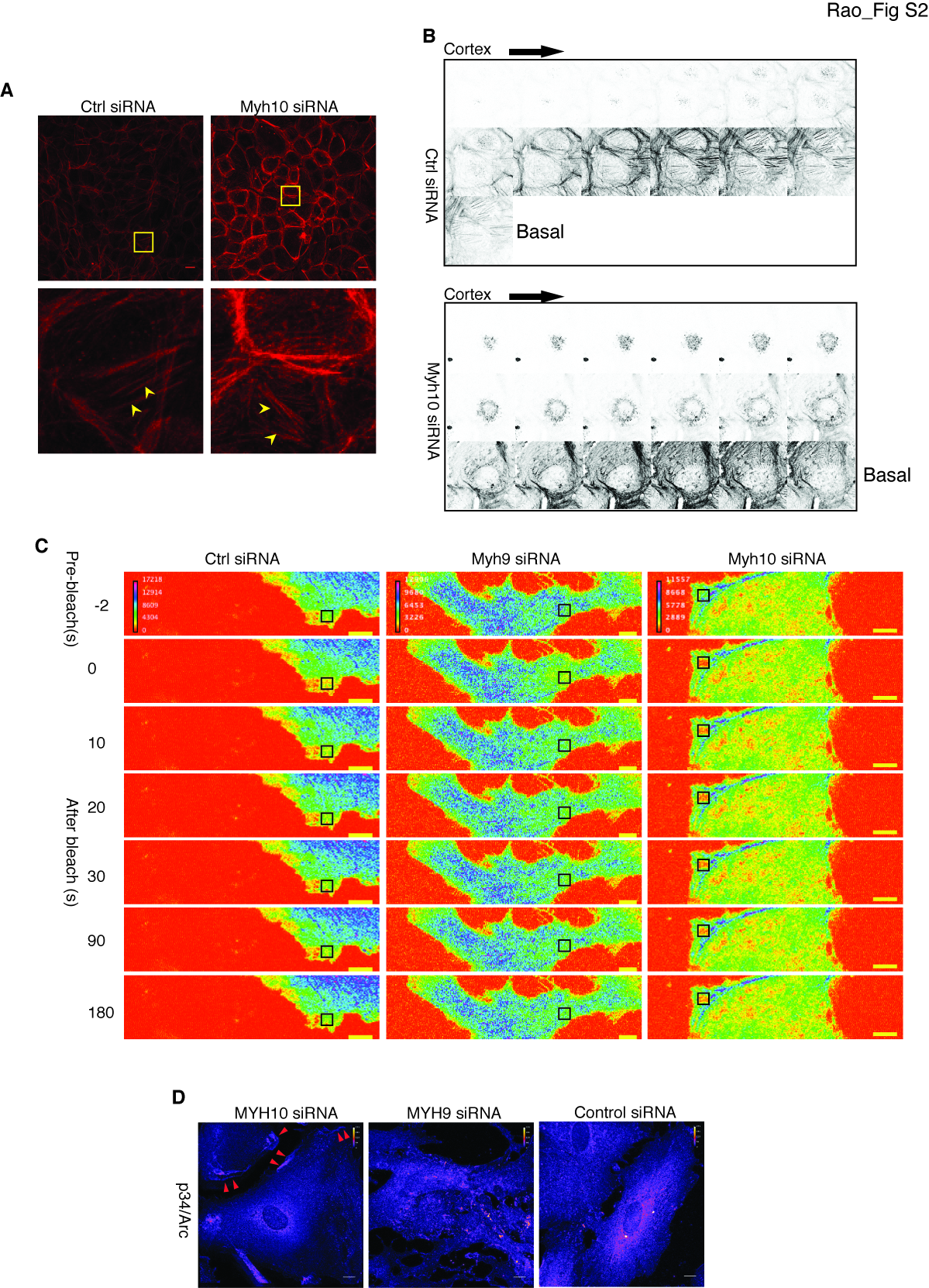

Supplement: S2 Figure — Myh10 knockdown stabilized F-actin network. (A) Myh10 knockdown stabilized F-actin network and did not disrupt stress fibers. Phalloidin-555 staining of F-actin network of control and Myh10 knockdown ARPE-19 cells. Yellow insets were magnified to allow better visualization of stress fibers (arrowheads). (B) Myh10 knockdown enhanced cortex actin network. Myh10 knockdown APRE-19 cells were stained with phalloidin-555 to label the actin network and imaged under a confocal microscope. Optical sections (0.3 µm) from cell cortex to cell bottom were shown in sequence. All images are shown as gray scale pictures. (C) Myh10 and Myh9 displayed opposite activity on actin dynamics. GFP-actin expressing (transient) ARPE-19 cells were transfected with control, Myh9 and Myh10 siRNA duplexes and subjected for FRAP analysis. Cells were photobleached near the cell edge and allowed to recover for 3 minutes during which images were acquired and selected time points were shown as spectrum images. Black insets indicates photobleached regions. Scale bar: 5 µm. (D) Myh10 knockdown enhanced branched actin network on the cell periphery. Myh9 and Myh10 knockdown RPE-Mchr1GFP cells were stained with rabbit anti-p34/Arc antibody to label branched actin network. Images were presented as spectrum. Red arrowheads indicate enhanced p34/Arc staining. (TIF) [file pone.0114087.s002.tif]

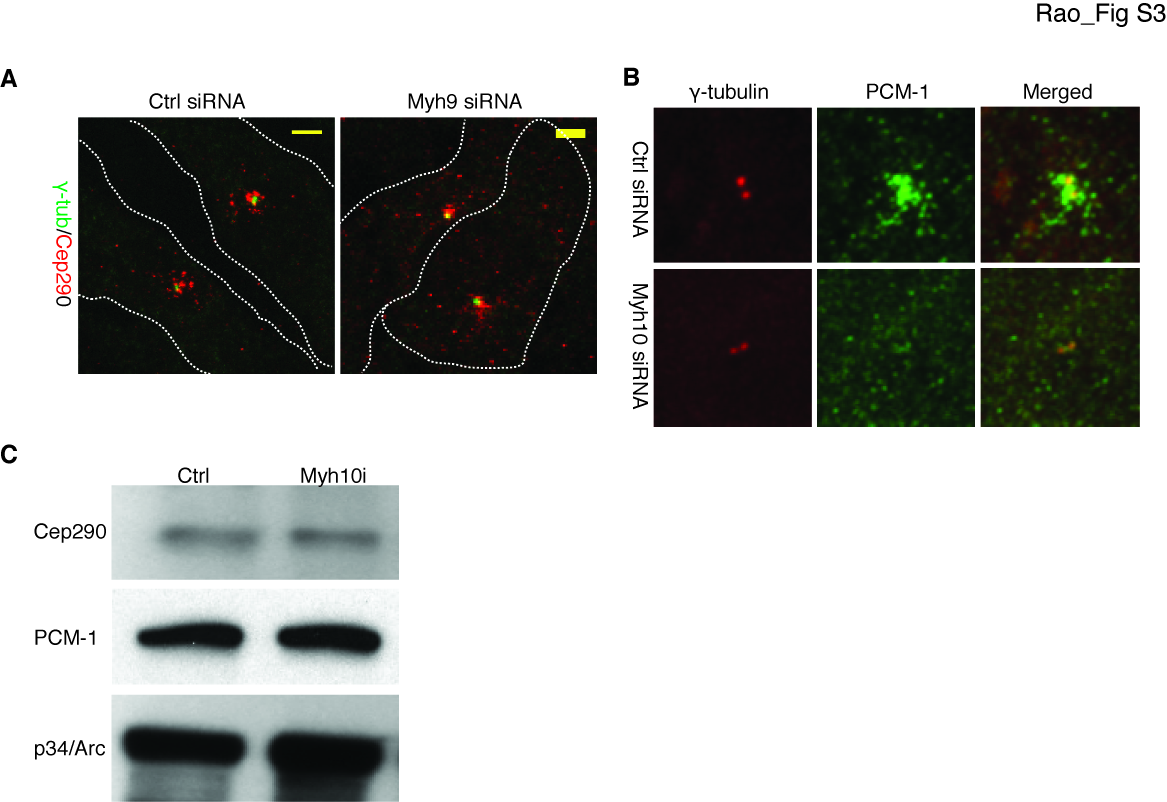

Supplement: S3 Figure — Myh10 knockdown specifically affected centriolar satellite organization. (A) Myh9 knockdown does not inhibit Cep290 centriolar satellite recruitment. Control and Myh9 knockdown RPE-Mchr1GFP cells were stained with Cep290 (red) and γ-tubulin (green). White dash lines outline individual cell morphologies. Scale bar: 5 µm. (B) Myh10 knockdown caused PCM-1 dispersion from centriolar satellites. Myh10-knockdown cells were stained with PCM-1 (green) and γ-tubulin (red). Representative images of centrosomal regions are shown. Control KD: upper panel; Myh10 KD: lower panel. (C) Myh10 knockdown does not affect Cep290 and PCM-1 protein abundance. Control (left column) and Myh10 KD (right lane) RPE-Mchr1GFP cells were collected for western blot analysis of Cep290 and PCM-1 protein levels. (TIF) [file pone.0114087.s003.tif]

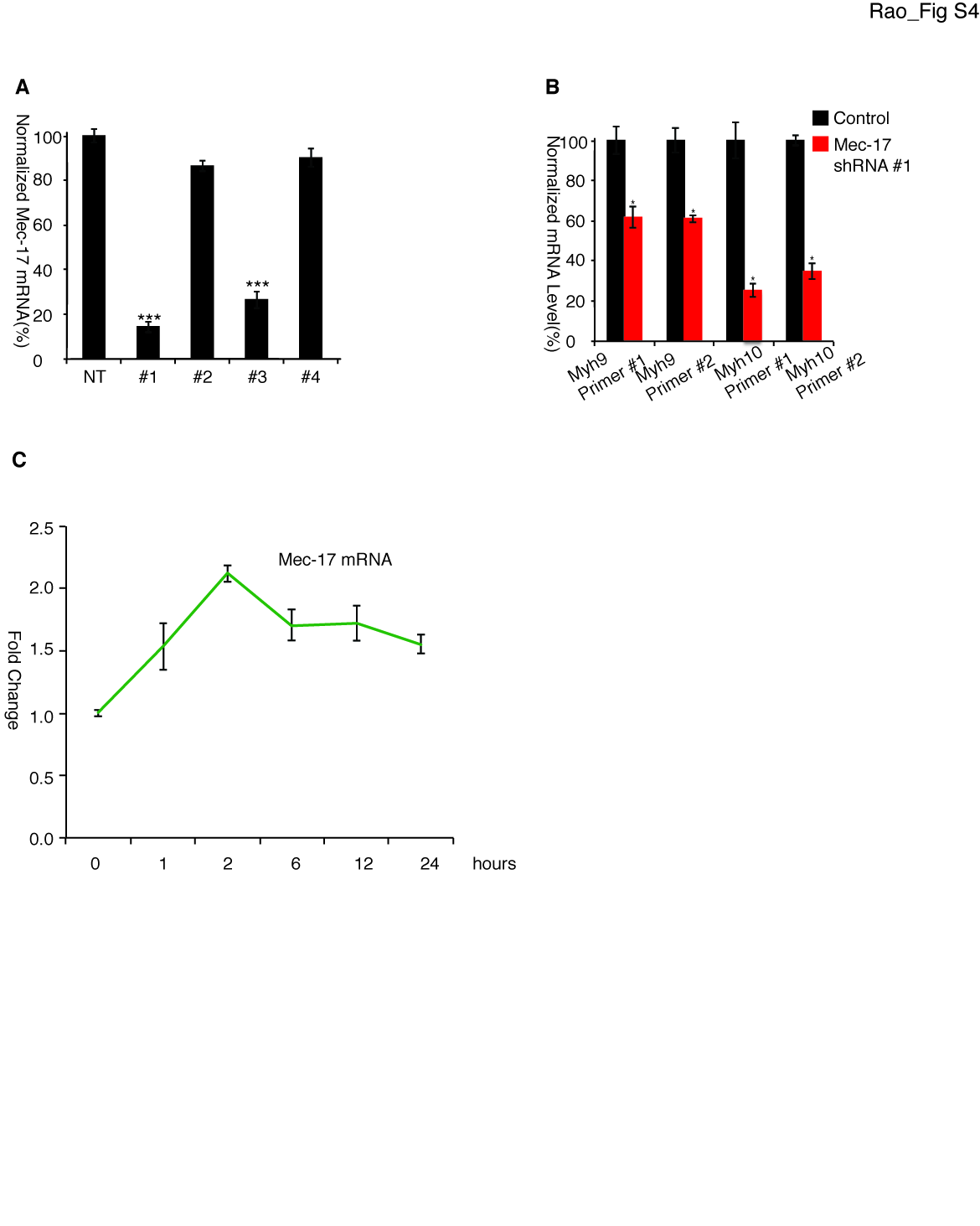

Supplement: S4 Figure — Myh10 expression is regulated by Mec-17 at the mRNA level during ciliogenesis. Q-PCR quantification of Mec-17 shRNA knockdown efficacy in ARPE-19 cells. ARPE-19 cells were infected with Mec-17 shRNA lentivirus supernatant and selected with 1 ug/ml puromycin for a week. mRNA was extracted and reverse transcribed for Q- PCR analysis. Mec-17 mRNA levels measured by Q-PCR were normalized to beta-actin control. The final results were presented as relative levels to non-target control shRNA-transduced cells. (A) Mec-17 knockdown suppressed Myh10 mRNA expression. ARPE-19 cells transduced with #1 Mec-17 shRNA lentivirus were harvested for Q-PCR analysis of Myh9 and Myh10 mRNA levels. Results are presented as relative levels to non-target control samples. (B) Mec-17 mRNA expression was up-regulated during ciliogenesis. RPE-Mchr1GFP cells were harvested after different time points of serum starvation. mRNA samples from different time points were subjected to Q-PCR analysis of Mec-17 mRNA levels. Results were presented as relative ratios to starting time point level. (TIF) [file pone.0114087.s004.tif]
